# Bacterial Cellulose Production from Industrial Waste and by-Product Streams

**DOI:** 10.3390/ijms160714832

**Published:** 2015-07-01

**Authors:** Erminda Tsouko, Constantina Kourmentza, Dimitrios Ladakis, Nikolaos Kopsahelis, Ioanna Mandala, Seraphim Papanikolaou, Fotis Paloukis, Vitor Alves, Apostolis Koutinas

**Affiliations:** 1 Department of Food Science and Human Nutrition, Agricultural University of Athens, Iera Odos 75, Athens 11855, Greece; E-Mails: eri.tsouko@gmail.com (E.T.); ladakisdimitris@gmail.com (D.L.); kopsahelis@upatras.gr (N.K.); imandala@aua.gr (I.M.); spapanik@aua.gr (S.P.); 2 UCIBIO-REQUIMTE, Department of Chemistry, Faculty of Science and Technology, New University of Lisbon, Caparica 2829-516, Portugal; E-Mail: c.kourmentza@fct.unl.pt; 3 Foundation of Research and Technology–Hellas, Institute of Chemical Engineering Sciences (FORTH/ICE-HT), Patras 26504, Greece; E-Mail: fpaluk@iceht.forth.gr; 4 LEAF-Linking Landscape, Environment, Agriculture and Food, Instituto Superior de Agronomia, Universidade de Lisboa, Lisboa 1349-017, Portugal; E-Mail: vitoralves@isa.ulisboa.pt

**Keywords:** *Komagataeibacter sucrofermentans*, bacterial cellulose, waste streams, biopolymer

## Abstract

The utilization of fermentation media derived from waste and by-product streams from biodiesel and confectionery industries could lead to highly efficient production of bacterial cellulose. Batch fermentations with the bacterial strain *Komagataeibacter sucrofermentans* DSM (Deutsche Sammlung von Mikroorganismen) 15973 were initially carried out in synthetic media using commercial sugars and crude glycerol. The highest bacterial cellulose concentration was achieved when crude glycerol (3.2 g/L) and commercial sucrose (4.9 g/L) were used. The combination of crude glycerol and sunflower meal hydrolysates as the sole fermentation media resulted in bacterial cellulose production of 13.3 g/L. Similar results (13 g/L) were obtained when flour-rich hydrolysates produced from confectionery industry waste streams were used. The properties of bacterial celluloses developed when different fermentation media were used showed water holding capacities of 102–138 g·water/g·dry bacterial cellulose, viscosities of 4.7–9.3 dL/g, degree of polymerization of 1889.1–2672.8, stress at break of 72.3–139.5 MPa and Young’s modulus of 0.97–1.64 GPa. This study demonstrated that by-product streams from the biodiesel industry and waste streams from confectionery industries could be used as the sole sources of nutrients for the production of bacterial cellulose with similar properties as those produced with commercial sources of nutrients.

## 1. Introduction

Cellulose is the most abundant natural polymer on Earth and it is found as a structural component, often bound to other polymers. Despite the fact that plant cellulose shares the same molecular formula (C_6_H_10_O_5_)_n_ with bacterial cellulose (BC), their physicochemical properties are different. BC is characterized by higher purity, due to the fact that it does not contain any hemicellulose or lignin, higher water holding capacity, hydrophilicity, degree of polymerization, mechanical strength, crystallinity, porosity, and a highly pure fiber network structure, compared to plant cellulose [1]. The enhanced mechanical properties of BC occur due to the uniform, continuous and nano-scalar network of cellulosic fibers. These properties are affected by various factors, such as the culture conditions, the microorganism and the fermentation media employed. Due to the versatile properties of this highly functional biopolymer, BC can be applied in numerous end-uses including scaffold for tissue engineering applications, wound healing applications, artificial skin in extensive burns, skin tissue repair, artificial blood vessels for microsurgery, sound transducing membranes, optically transparent composites, in paper manufacturing [2], and in the food industry as a thickening and stabilizing agent [3].

Despite its enormous potential in various applications, the high cost of BC production is the main drawback that hinders industrial implementation. The utilization of industrial wastes and by-product streams as fermentation media could improve the cost-competitiveness of BC production. In recent years, many studies have focused on developing cost-effective fermentation media for BC production, such as konjac powder [4], fruit juices [5], maple syrup [6], thin stillage [7], wheat straw [8], spruce hydrolysate [9], crude glycerol from biodiesel production processes, grape bagasse [10], waste water of candied jujube [11], and acetone-butanol-ethanol fermentation wastewater [12].

The worldwide biodiesel production was more than 431 thousand barrels per day in 2012, representing an average increase of 39% over the last five years [13]. Biodiesel production in Europe represents approximately 40% of the worldwide production capacity [14]. Biodiesel is currently produced mainly from oilseeds leading to the generation of significant quantities of by-product streams, namely crude glycerol and oilseed meals. Crude glycerol is produced as a 10% (*w*/*w*) by-product stream during transesterification of triglycerides with an alcohol, most frequently methanol. Oilseed meals constitute the solid residues that remain after the extraction of oil from the oilseeds. Sunflower meal (SFM) is used for biodiesel production in southern European countries. The worldwide production of sunflower meal was 16.04 million t in 2014/2015 with 25% of the total production being cultivated in the European Union [15].

Food losses and wastes refer to the decrease of food along the food supply chain starting from initial agricultural production up to final human consumption. One-third of food produced for human consumption is lost or wasted globally, which amounts to about 1.3 billion t per year [16]. Flour-rich waste (FRW) streams are mainly generated during the manufacturing process of bread and confectionery products, disposal by consumers and catering services or as end-of-date products. FRW contain significant quantities of starch, protein, and various micro-nutrients, which could be used as fermentation feedstock.

This study focuses on the evaluation of bacterial cellulose production from by-product streams generated by sunflower-based biodiesel industries and waste streams generated by the confectionery industry. Furthermore, the physical and mechanical properties of the bacterial celluloses produced from different fermentation media were determined.

## 2. Results and Discussion

### 2.1. Assessment of Carbon Sources for Bacterial Cellulose Production

**Figure 1:**
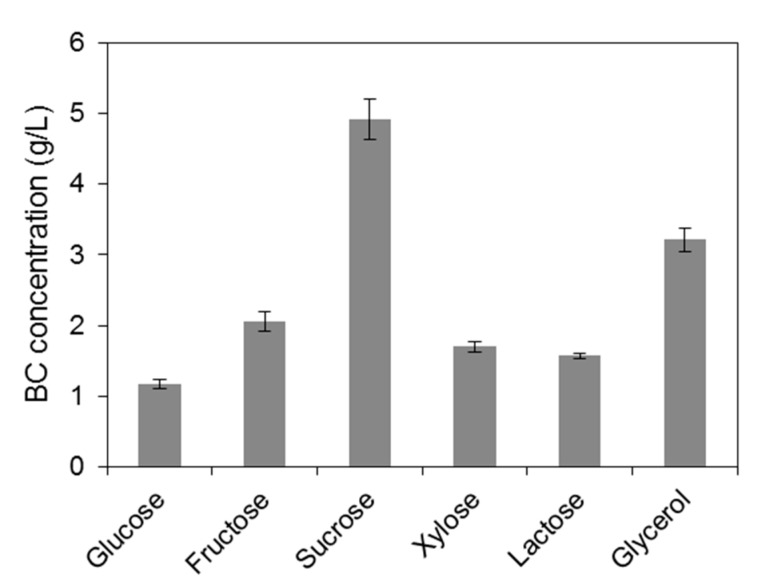
Bacterial cellulose production achieved when various commercial sugars and crude glycerol were used in shake flask fermentations.

Various commercial sugars (xylose, lactose, glucose, sucrose, fructose) and crude glycerol were initially evaluated using the same fermentation media (Figure 1) in order to determine their efficiency on BC production by the bacterial strain *Komagataeibacter sucrofermentans* DSM 15973. Xylose and lactose were not efficiently metabolized by the bacterial strain resulting in low BC production of 1.7 and 1.6 g/L, respectively. Ishihara *et al.* [17] reported that xylose is oxidized to D-xylonic acid by *Αcetobacter xylinus* IFO 15606 leading to xylonate accumulation in the broth and low pH values that inhibit bacterial growth and BC production. In the case of lactose, the low BC concentration obtained can be attributed to the fact that members of the Acetobacteraceae family, to which *A. xylinus* belongs, do not possess the gene that encodes β-galactosidase, the enzyme responsible for the hydrolysis of lactose [18]. Utilization of glucose resulted in lower BC production (1.2 g/L) than literature-cited studies (up to 4.1 g/L) [19,20]. The utilization of increasing glucose concentrations may lead to gluconic acid production, as by-product during cellulose biosynthesis, resulting in gradually decreasing pH values and lower glucose to cellulose production yields [21]. Dahman *et al.* [22] reported that the highest BC concentration (5.65 g/L) was achieved in a fructose-based medium, which is higher than the BC concentration achieved in this study when fructose was used as carbon source (2.06 g/L). Crude glycerol and commercial sucrose led to the highest BC concentrations of 3.2 and 4.9 g/L, respectively. Generally, these results are in good agreement with literature-cited publications [22,23,24].

### 2.2. Evaluation of Different Free Amino Nitrogen Concentrations for BC Production

**Figure 2:**
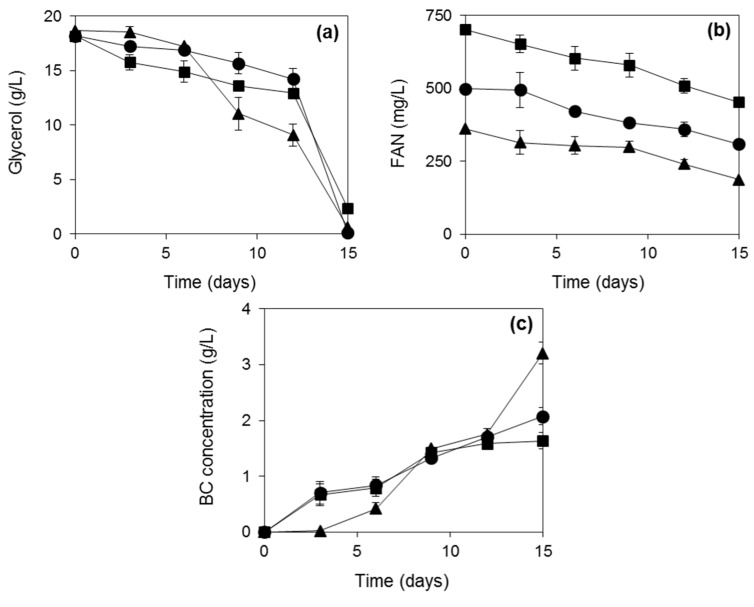
Glycerol (**a**) and FAN (**b**) consumption and BC (**c**) production using crude glycerol as carbon source and yeast extract with peptone as nitrogen sources at different initial FAN concentrations. The fermentation duration was 15 days. (▲), 360 mg/L FAN; (●), 500 mg/L FAN; (■), 700 mg/L FAN.

Subsequent experiments focused on the evaluation of the nitrogen content on BC production when commercial yeast extract and peptone were used in shake flask fermentations with crude glycerol as carbon source. It was observed that crude glycerol was consumed almost entirely (Figure 2a) in all experiments where yeast extract together with peptone were used as nitrogen sources. However, BC production decreased (Figure 2c) with increasing free amino nitrogen (FAN) concentration (Figure 2b). When the lowest FAN concentration was used (360 mg/L), the final BC concentration was 3.2 g/L. The BC produced with 500 and 700 mg/L initial FAN concentration were 35% and 48% lower than the BC concentration achieved with the lowest FAN concentration. Figure 2b shows that the FAN concentration consumed in the three cases was 174, 189 and 249 mg/L respectively at the end of fermentation when the carbon source was depleted from the medium. BC production occurs simultaneously with bacterial growth. Therefore, high nitrogen concentrations may favor cell growth at the expense of BC production.

### 2.3. BC Production from Biodiesel and Confectionery Industry Side Streams

**Figure 3:**
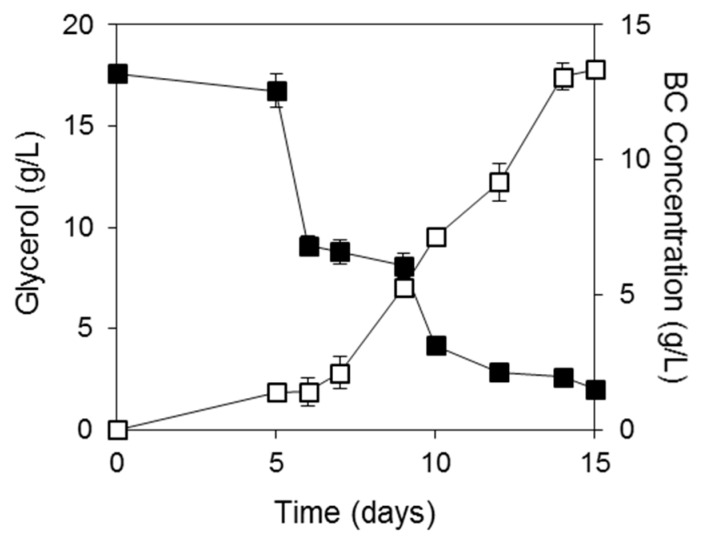
Crude glycerol consumption and BC production using SFM hydrolysates and crude glycerol as fermentation media. (■), Glycerol; (□), BC.

Crude glycerol and SFM hydrolysates were subsequently evaluated as feedstocks for BC production (Figure 3). The initial FAN concentration employed was 350 mg/L. The sunflower meal hydrolysate was produced by a two-stage bioprocess involving solid-state fermentation of *Aspergillus oryzae* for the production of crude enzyme consortia that were subsequently employed for the hydrolysis of untreated sunflower meal [25]. The SFM hydrolysate does not contain any sugars and glycerol was the only carbon source provided in the fermentation medium. BC production was enhanced 4 fold (13.3 g/L after 15 days) when biodiesel industry by-products were used in comparison to the use of commercial nutrient supplements and crude glycerol. The glycerol to BC conversion yield achieved was 0.8 g BC per g of consumed glycerol and the productivity was 0.89 g/L/day. Glycerol consumption was initiated approximately after the 5th day, while BC production increased significantly after the 6th day of fermentation.

**Figure 4:**
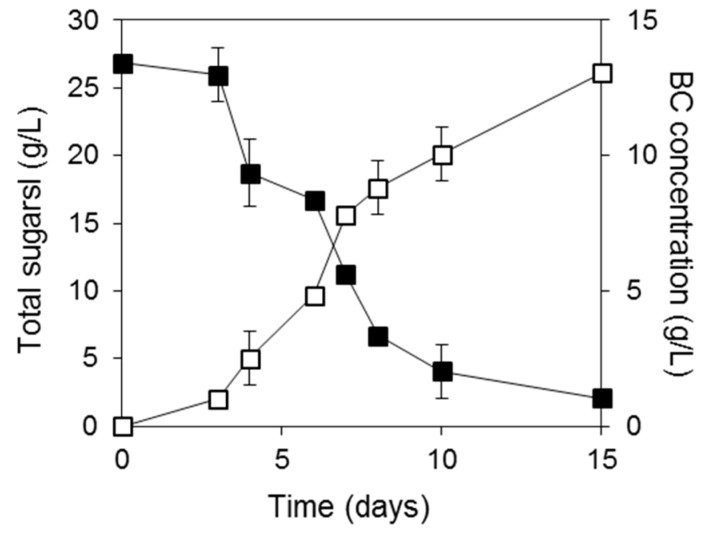
Total sugar consumption and BC production when FRW hydrolysates were used as fermentation media. (■), Total sugars; (□), BC.

The production of FRW hydrolysates has been described by Tsakona *et al.* [26]. Crude enzymes were initially produced via solid-state fermentation of *Aspergillus awamori* cultivated on wheat flour milling by-products. An enzyme-rich aqueous extract was used for the hydrolysis of starch and protein that are contained in FRW. This nutrient-rich hydrolysate was subsequently used as fermentation medium for BC production. The original FRW contained starch and sucrose, which were both hydrolyzed into the respective monomers, *i.e.*, glucose and fructose, because the fungal strain *A. awamori* produces both glucoamylase and invertase during solid-state fermentation. The FRW hydrolysates used contained 0.8 g/L of sucrose, 18.6 g/L of glucose, 7.5 g/L of fructose and a FAN concentration of around 350 mg/L. Figure 4 presents the consumption of total sugars and the production of BC in a 15 days culture. The final BC concentration produced was 13 g/L. When commercial sugars were used, the highest BC production was achieved with commercial sucrose. FRW hydrolysates led to the production of 2.6 times higher BC concentration than commercial sucrose and nutrient supplements. The sugar to BC conversion yield was 0.53 g/g and the productivity was 0.87 g/L/day.

Table 1 presents the BC production achieved in literature-cited publications when various renewable resources were used. In these studies, the BC concentration achieved was in the range of 0.3–18 g/L. The highest BC concentration of 18 g/L was achieved when the bacterial strain *Acetobacter xylinum* KJ1 was cultivated on saccharified food wastes in a 30 L bioreactor [27].

**Table 1:** BC production using various natural resources based on literature-cited publications.

Strain	Fermentation Mode	Carbon Source	BC (g/L)	Productivity (g/L/d)	Reference
*Acetobacter aceti* subsp. *xylinus* ATCC 23770	Static batch fermentations	Konjac powder	2.1	0.26	[4]
*A. xylinum* NBRC 13693	Static batch fermentations	Fruit juices	5.9	0.42	[5]
*A. xylinum* BPR 2001 (ATCC 700178)	Agitated batch cultures (135 rpm)	Maple syrup	1.5	0.07	[6]
*Gluconacetobacter xylinus* BCRC 12334	Static batch fermentations	Thin stillage from rice wine distillery	10.4	1.48	[7]
*G. xylinus* ATCC 23770	Static batch fermentations	Wheat straw hydrolysates	8.3	1.18	[8]
*G. xylinus* ATCC 23770	Static batch fermentations	Spruce hydrolysates	8.2	0.59	[9]
*G. xylinus* NRRL B-42	Static batch fermentations	Glycerol from biodiesel production process	10	0.71	[10]
		Grape bagasse from wine production	8	0.57	
*G. xylinum* CGMCC 2955	Static batch fermentations	Wastewater of candied jujube processing industry	2.2	0.37	[11]
*G. xylinus* CH001	Static batch fermentations	Acetone-butanol-ethanol fermentation wastewater	1.3	0.19	[12]
*A. xylinum* KJ1	Static cultures in 30 L fermentor	Saccharified food wastes	18	3.6	[27]
	Agitated culture in 10 L jar fermentor		16.8	3.36	
*G. xylinus* ATCC 23770	Static batch fermentations	Cotton cloth hydrolysates	10.8	0.77–1.54	[28]
*G. hansenii* CGMCC 3917	Static batch fermentations	Waste beer yeast hydrolysates	7	0.5	[29]
*A. xylinum* KJ1	50 L spherical type bubble column bioreactor	Saccharified food wastes	5.6	1.87	[30]
*G. medellinensis*	Static batch fermentations	Pineapple waste and sugar cane juice	4	0.3	[31]
	Agitated batch fermentations		0.8	0.11	
*G. sacchari*	Static batch fermentations	Grape skins aqueous extract	0.6	0.15	[32]
		Industrial hardwood spent sulfite liquor	0.3	0.07	

In the following sections, the properties of the BCs produced from biodiesel industry by-products (BC_2_) and confectionery industry wastes (BC_4_) were determined and compared with the properties of BC produced with either crude glycerol combined with the Hestrin and Schramm (HS) medium after the removal of glucose (BC_1_) or with the HS medium alone (BC_3_).

### 2.4. Water Holding Capacity (WHC)

The WHC (water holding capacity) is considered one of the most important physical characteristics of bacterial cellulose pellicles regarding biomedical application of BC as wound dressing material. The variations between the WHC are related to the porosity and surface area of each BC. The greater the surface area and the pore size the larger the amount of water penetrated and trapped in the BC matrix. Table 2 shows the WHC of the different BC produced by different media. The WHC of BC_1_ is the highest (138 ± 9 g/g) of all BC samples followed by BC_3_ (131 ± 4 g/g), BC_2_ (124 ± 5 g/g) and BC_4_ (102 ± 6 g/g). The utilization of crude glycerol together with the HS medium after the removal of glucose led to the production of BC_1_ with the highest WHC. The BC_2_ and BC_4_ produced from the renewable resources used in this study demonstrated also WHC higher than 99%. The WHC of the BCs produced in this study are in good agreement with literature-cited publications [33] and sometimes even higher [34].

**Table 2:** Properties of bacterial cellulose samples.

Properties	BC_1_	BC_2_	BC_3_	BC_4_
Stress at break [σ] (MPa)	139.5 ± 12.6	79.8 ± 7.6	94.5 ± 8.2	72.3 ± 6
Elongation at break [ε] (%)	8.5 ± 0.2	7.1 ± 0.0	9.2 ± 0.4	7.05 ± 0.02
Young’s modulus [Ε] (GPa)	1.64 ± 0.2	1.13 ± 0.11	1.02 ± 0.09	0.97 ± 0.05
Crystallinity index [CrI] (%)	88	74	81	89
Mean crystallite size [CrS] (nm)	5.9	6.4	6.1	5.7
Intrinsic viscosity [η] (dL/g)	9.3	7.5	4.7	6.2
Molecular weight [*M*_W_] (10^6^ g·mol^−1^)	0.43	0.39	0.31	0.35
Degree of polymerization [DP]	2672.8	2391.2	1889.1	2176.1
Water holding capacity [WHC] (g·water/g·dry BC)	138 ± 9	124 ± 5	131 ± 4	102 ± 6

### 2.5. Effect of Different Culture Media on the Intrinsic Viscosity and Degree of Polymerization

Intrinsic viscosity, (η), is a property that determines the hydrodynamic volume occupied by the polymeric macromolecule in a specific solvent. Therefore, it depends on the type of solvent used, the molecular mass of the polymer, and the nature of the chemical bond between the individual sugars. In addition, higher viscosities are related to greater polysaccharide chain stiffness, *i.e.*, β*-*(1,4) bonds render stiffer chains than α*-*(1,4) or β*-*(1,3) linkages [35].

According to the results presented in Table 2, the intrinsic viscosity values obtained for the different types of bacterial cellulose produced ranged between 4.7–9.3 dL/g. Typical intrinsic viscosity values of polysaccharides may vary from 1 to 20 dL/g [36].

The degree of polymerization (DP) ranged between 1889.1–2672.8. In the case where crude glycerol was used together with the HS medium after the removal of glucose, the BC_1_ produced was characterized by the highest DP (2672.8). The BC_2_ produced from biodiesel industry by-products demonstrated the second highest DP (2391.2), whereas the BC_4_ produced from confectionery industry waste streams demonstrated the third highest DP (2176.1). The DP of bacterial cellulose usually ranges between 2000 and 8000 [33,37].

Shi *et al.* [38] reported DP values of 1380, 1361, and 2301 when different BC were produced by *G. intermedius* BC-41 cultivated on glycerol, xylose and glucose, respectively. Keshk [39] reported BC production with DP in the range of 1748 to 2378 from four strains of *G. xylinus* ATCC 10245, IFO 13693, 13772, and 13773 using either HS medium or HS medium supplemented with 1% lignosulfonates.

### 2.6. Mechanical Properties

**Figure 5:**
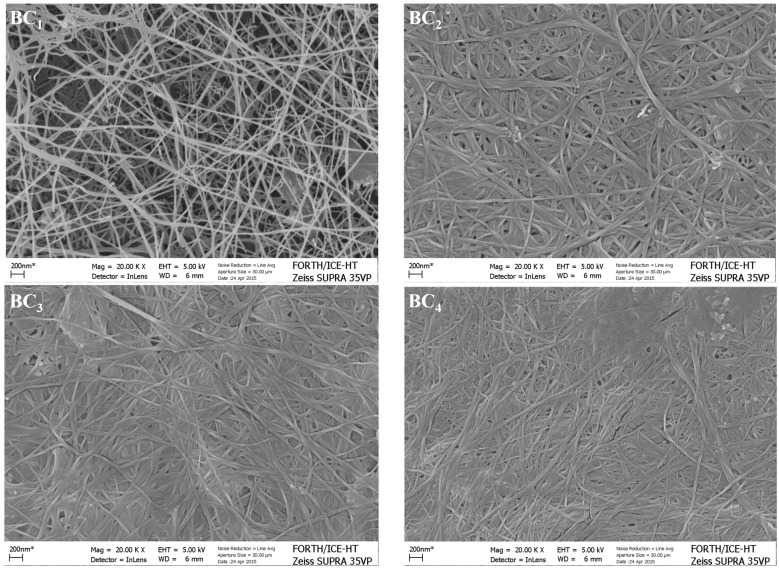
SEM images of BC samples that were produced using different fermentation media. BC_1_, crude glycerol combined with glucose-free HS medium; BC_2_, crude glycerol combined with SFM hydrolysate medium; BC_3_, HS medium; and BC_4_, FRW hydrolysate medium (all micrographs correspond to 20k magnification).

Most of the studies concerning the mechanical properties of BC are carried out using hydrated pellicles. In the present study, the mechanical properties of lyophilised bacterial cellulose films were evaluated. According to the obtained results, sheets are characterized by strains between 7.1%–9.2% ± 0.4% before brittle failure. Tensile stress at break and Young’s modulus ranged from 72.3 ± 6 to 139.5 ± 12.6 MPa and 0.97 ± 0.05 to 1.64 ± 0.20 GPa, respectively. BCs with higher DP are expected to have greater mechanical strength [40]. BC_1_ showed the highest tensile strength (139.5 ± 12.6 MPa) followed by BC_3_ (94.5 ± 8.2 MPa), BC_2_ (79.8 ± 7.6 MPa) and BC_4_ (72.3 ± 6 MPa). Differences between the BC samples can be also attributed, as it can be observed by Scanning Electron Microscopy (SEM) micrographs (Figure 5), to their different fibrillar orientation and fibril width. For example, BC_1_ is characterized by a more organized fibril network, compared to the other BC samples, and a fibril width as high as the one observed in the case of BC_3_.

George *et al.* [41] reported a tensile strength of 43.68 MPa in the case of sodium hydroxide-treated BC produced by *Acetobacter xylinum* using a medium rich in sucrose and yeast extract. In another study, when *G. hansenii* was cultivated in a medium consisting of glucose, yeast extract and peptone, tensile strength reached 76.7 MPa [42], while the tensile strength of BC produced by *Acetobacter xylinum* grown on HS medium was reported to be 120 MPa [43]. The in results reported this study are in good agreement with the literature, but in general the values concerning the parameters of the mechanical properties vary according to the methodology followed. Furthermore, several factors affect the mechanical properties of bacterial cellulose films, such as the incubation period, failure stress under uniaxial or biaxial tension [44], the drying technique applied [45], the pressure applied to the pellicle before drying [46], as well as the concentration of NaOH used for BC treatment [41].

### 2.7. XRD Analysis

**Figure 6:**
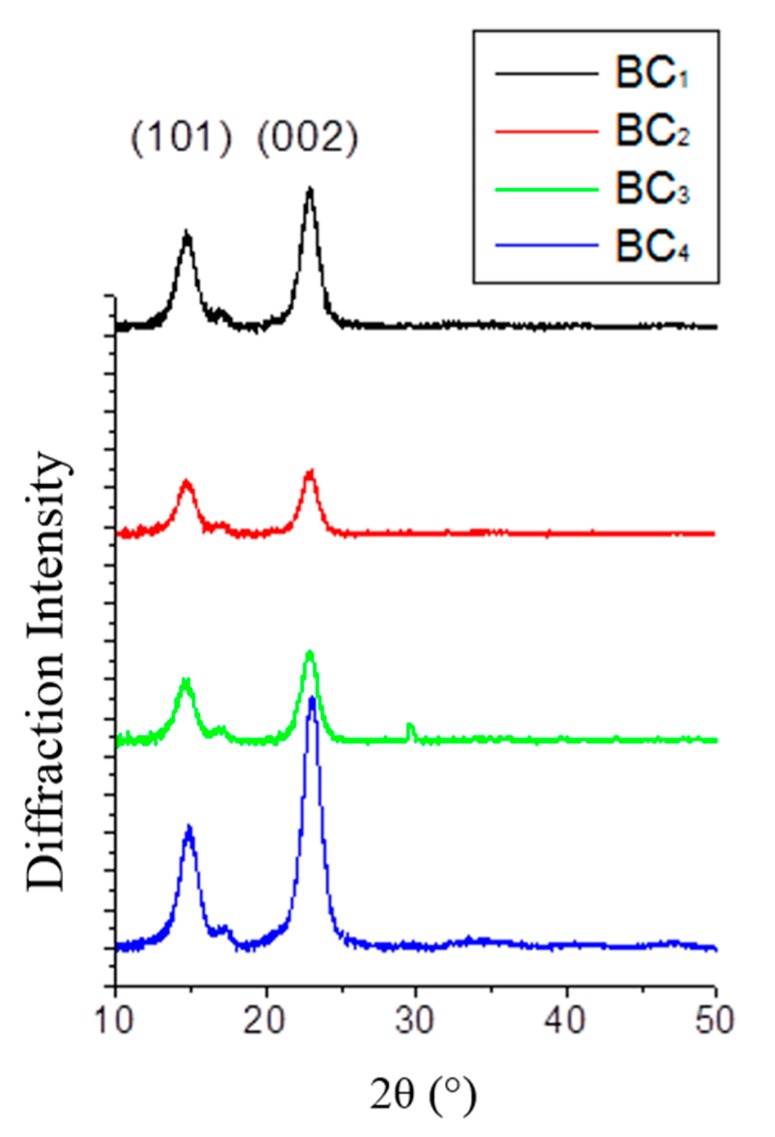
XRD diffraction patterns of bacterial cellulose samples.

Figure 6 illustrates the X-ray diffraction patterns of the four samples of bacterial celluloses. The diffraction diagrams of the BC produced in different media revealed three characteristic diffraction peaks indicating the presence of both *I*_α_ and *I*_β_ crystal cellulose. According to the data obtained from the XRD analysis the crystallinity and crystallite size were calculated and summarized in Table 2. The highest crystallinity percentages were identified in BC_1_ (88%) and BC_4_ (89%), which were produced from crude glycerol combined with glucose-free HS medium and confectionery industry waste streams, respectively. The BC_2_ produced from biodiesel industry by-products exhibited the lowest crystallinity percentage (74%). The BC_3_ produced using the HS medium was characterized by a crystallinity index of 81%. The average particle crystallite size ranged between 5.7–6.4 nm.

In general, BC shows high degree of crystallinity. A CrI value of 95.2% in the case of bacterial microcrystalline cellulose produced by *G. hansenii* ATCC 10821 was determined by the XRD peak height method [47]. Lu and Jiang [48], who used *A. xylinum* strain Y22 and fermentation medium that contained sucrose supplemented with yeast extract and peptone under static cultivation, reported the production of BC with a CrI of 90.5% and particle size of 11.9 nm. Cultivation of *G. xylinus* PTCC 1734 on mannitol and sucrose resulted in the production of bacterial cellulose that was characterized by CrI and CrS in the range of 46.7%–65.5% and 5.8–6.4 nm, respectively [49].

### 2.8. BC Morphology Characterization

Morphological characterization of different BC samples was carried out using SEM. Selected micrographs of the surface of different BC samples are shown in Figure 5. All samples are characterized by their fibrillar, interwoven nano-sized structure. BC_1_ is characterized by a circular-pore structure formed by ribbon fibrils networks. BC_2_, BC_3_, and BC_4_ showed a more disorganized layered structure, which was formed by random overlaying structure of rounder fibrils, compared to BC_1_.

The use of different media seems to have an effect on fibril width. The fibril width for samples BC_1_ and BC_2_ is characterized by a higher variation, 33 ± 5 and 27 ± 10 nm, respectively, whereas samples BC_3_ and BC_4_ showed lower variations (34 ± 2 and 27 ± 4 nm, respectively).

According to the surface morphology, it can be seen that BC_1_ showed a relatively less dense and more porous structure. On the other hand, a large number of pores of various, but smaller sizes, can be observed in the cases of BC_2_ and BC3 that showed high water holding capacities (124 ± 5 and 131 ± 4 g·water/g·dry weight, respectively). The highest WHC value was obtained in the case of BC_1_ (138 ± 9 g·water/g·dry weight). BC4 seems to be formed by a more dense and multi-layered fibril network.

The thickness of fibrils, together with DP and CrI, are among the factors that affect the water retention of bacterial cellulose. More water is retained due to the larger surface area provided by thinner and longer fibers [50]. The retention of water in BC occurs via physical entrapment at the surface and within the particles that contain reticulated fibrils [50].

## 3. Experimental Section

### 3.1. Raw Materials

Crude glycerol and SFM were kindly provided by the biodiesel producer Pavlos N. Pettas S.A. (Patras, Greece), while FRW streams were supplied by Jotis SA (Athens, Greece), a Greek confectionery industry that produces a wide range of confectionery products and food for infants. The composition of crude glycerol and SFM was presented by Kachrimanidou *et al.* [25].

### 3.2. Microorganisms

The fungal strains *Aspergillus oryzae* and *Aspergillus awamori* were kindly provided by Colin Webb (University of Manchester, Manchester, UK). Strains were stored at 4 °C in agar slopes, containing 50 g/L wheat milling by-products and 20 g/L agar in the case of *A. awamori* and 30 g/L SFM, 20 g/L wheat milling by-products and 20 g/L agar in the case of *A. oryzae*. Both strains were used for the production of crude enzyme consortia through solid-state fermentation, which were subsequently used in hydrolysis experiments. More specifically, *A. oryzae* (producing mainly proteolytic enzymes) was involved in the SFM hydrolysis experiments, while *A. awamori* (producing mainly glucoamylase) was involved in FRW hydrolysis.

The bacterial strain *Komagataeibacter sucrofermentans* DSM 15973 was used for the production of bacterial cellulose. Bacterial stock cultures were maintained at 4 °C in petri dishes. Liquid media for inocula preparation constituted of 20 g/L glucose, 5 g/L peptone, 5 g/L yeast extract, 2.7 g/L Na_2_HPO_4_, 1.15 g/L citric acid (HS media), while the pH of the medium was adjusted to 6. Solid media for bacterial stock cultures contained also 20 g/L agar-agar.

### 3.3. Solid State Fermentation

Solid-state fermentations were conducted in 250 mL Erlenmeyer flasks at 30 °C. A quantity of 5 g of SFM (for *A. oryzae*) or wheat milling by-products (for *A. awamori*) was added in each flask that was autoclaved at 121 °C for 20 min. Subsequently, the solids in each flask were inoculated with a fungal spore suspension (10^6^ spores/mL) that was also used to adjust the moisture content of the substrate at 65% (*w*/*w*, db). Sporulation and inocula preparation for solid-state fermentations have been presented by Kachrimanidou *et al.* [51]. Each fermentation lasted for 48 h.

### 3.4. Production of SFM and FRW Hydrolysates

Remaining solids after SSF were macerated using a kitchen blender and then mixed with 500 mL deionized water in 1 L Duran bottles, which contained 50 g/L SFM or 70 g/L FRW. The Duran bottles were placed in a water bath for 24 h at 45 °C in the case of SFM hydrolysis and 55 °C in the case of FRW hydrolysis. Mixing of the suspension was achieved with magnetic stirrers. After the end of the hydrolysis, SFM or FRW hydrolysates were centrifuged at 10,000× *g* and 4 °C for 15 min to separate the solids and the supernatant was filter-sterilized through a 0.22 μm filter unit (Polycap AS, Whatman™ Ltd., Buckinghamshire, UK).

### 3.5. Fermentative Production of Bacterial Cellulose

All fermentations were conducted under sterile conditions in 250 mL Erlenmeyer flasks with 50 mL working volume at 30 °C. The volume of the inoculum was 10% (*v*/*v*) and the pH value of the broth was adjusted to 6 using 5 M NaOH. The flasks were initially incubated at 100 rpm for 1–2 days and then statically until the end of the fermentation (15 days).

The first series of fermentations was carried out using modifications of the HS media (substitution of glucose with other carbon sources, such as xylose, sucrose, fructose, lactose and crude glycerol). The initial FAN concentration in all cases was ~350 mg/L. Moreover, in the case of BC production using crude glycerol as carbon source, the effect of different initial FAN concentration was evaluated. More specifically, concentrations of 5, 7.5 and 10 g/L of yeast extract and peptone were used in order to achieve initial FAN concentrations of 360, 500 and 700 mg/L, respectively. SFM hydrolysates were diluted with tap water in order to adjust the initial FAN concentration. SFM hydrolysates (together with crude glycerol) and FRW hydrolysates were applied as fermentation media for the production of BC.

### 3.6. Characterization of BC Samples

Bacterial cellulose samples obtained in the different experiments were characterized in terms of water holding capacity, viscosity, degree of polymerization, crystallinity and mechanical strength. BC morphology characterization was carried out using Scanning Electron Microscopy (SEM). The BC samples were designated as: BC_1_, produced using crude glycerol combined with HS medium (excluding glucose); BC_2_, produced using the crude glycerol-SFM hydrolysate medium; BC_3_, using the HS medium alone; BC_4_, using FRW hydrolysate media.

#### 3.6.1. Water Holding Capacity (WHC)

The water holding capacity of the samples was measured using the shake method [52]. BC samples were removed from the storage container using tweezers. The samples were shaken twice, to remove excess surface water, and then weighed. Samples were left to dry at room temperature for 48 h and subsequently dried for 12 h at 60 °C in order to remove completely the water content. The water holding capacity was calculated using the following formula:

(1) $$\text{Water Holding Capacity}=\frac{\text{Mass of water removed during drying }\left(\text{g}\right)}{\text{Dry weight of bacterial cellulose }\left(\text{g}\right)}$$

#### 3.6.2. Intrinsic Viscosity and Degree of Polymerization (DP)

Lyophilized BC was weighed and dissolved in Copper(II)-ethylenediamine solution (CED) and the specific concentration (c) was determined. The relative viscosity (η_r_) and specific viscosity (η_sp_) of BC in copper-ethylenediamine solution were measured using an Ubbelohde viscometer (Schott Micro-Ubbelohde Viscometer IIc, Mainz, Germany) immersed in a water bath at constant temperature, 25 ± 0.5 °C, and then calculated according to the following equations:

(2) $${\text{η}}_{\text{r}}=\frac{t}{t_{0}}$$

(3) $${\text{η}}_{\text{sp}}=\;{\text{η}}_{\text{r}}-1$$

where *t*_0_ is the flow time of the solvent alone and t is the flow time of the bacterial cellulose solution at each specific concentration. The intrinsic viscosity (η) of the solutions was determined using the general formula:

(4) $$\text{η}=\;\frac{\sqrt{2\left({\text{η}}_{\text{sp}}-\ln{\text{η}}_{\text{r}}\right)}}{c}$$

The average molecular weight (*M*) was determined by using the Mark-Houwink empirical equation:

(5) $$\text{η}=Κ{Μ}^{\text{α}}$$

The values of the Mark-Houwink parameters, *K* and α, for the particular polymer-solvent system were obtained from the Polymer Data Handbook [53]. Finally, the degree of polymerization (*DP*) was calculated as the average molecular weight (*M*) of the polymer divided by the molecular weight of an anhydrous glucose monomeric unit:

(6) $$DP=\;\frac{M}{162}$$

#### 3.6.3. X-ray Diffraction Analysis (XRD)

The X-ray diffraction (XRD,) technique was employed in order to determine the crystallinity index and crystallite size of the bacterial cellulose samples. XRD patterns of the lyophilized films were recorded using a Bruker D8 Advance diffractometer (Bruker, Carlsruhe, Germany) at the CuKα radiation wavelength (λ = 1.54 Å), generated at a voltage of 40 kV and a filament emission of 40 mA. Samples were scanned in a 2θ range between 5° and 90°. The scan speed was 0.5°·min^−1^ with a step size of 0.02°, and the scans were collected from 2θ  =  5° to 50°. Data were obtained by the EVA 15.0 DIFFRAC PLUS software package attached to the XRD instrument. The crystallinity index (*CrI*) was calculated using the peak intensity method:

(7) $$CrI=\frac{I_{002}-I_{AM}}{I_{002}}\;\times \;100$$

where, *I*_002_ is the maximum intensity of the (002) lattice diffraction and *I*_AM_ is the intensity diffraction at 2θ (≈23°).The mean crystallite (*CrS*) size was calculated from the X-ray line broadening of the (002) diffraction peak according to Scherrer’s Equation:

(8) $$CrS=\;\frac{\text{kλ}}{\text{βcosθ}}$$

where, k is the shape factor (0.89), β is the wavelength of X-ray radiation (1.54 Å), β is the full width at half maximum height and θ is the Bragg’s angle.

#### 3.6.4. Mechanical Strength

The tensile properties of bacterial cellulose membranes were examined using a TA-Xtplus texture analyzer (Stable Micro Systems, Surrey, UK). The tests were performed at room temperature, *T* = 22.0 ± 2.0 °C. The lyophilized samples were mounted between an upper and a lower clamp and force was applied from the upper clamp with a speed of 0.5 mm/s in tension mode. Tensile stress at break, σ, was calculated as the ratio of the tensile load *F* applied to the specimen to the original cross-sectional area *A*_o_ of the samples. Elongation at break, ε, was determined as the ratio of the increase of the specimen gauge length to its original gauge length (Δ*L*/*L*_o_). Young’s modulus, or modulus of Elasticity, *E*, was calculated by the slope of the stress-strain curve at the linear region. Three film replicates were analyzed.

#### 3.6.5. Scanning Electron Microscopy (SEM)

The surface and morphology of BCs were studied by Scanning Electron Microscopy (Zeiss Supra 35VP, Carl Zeiss, Oberkochen, Germany). The freeze-dried samples were coated with gold and examined at an accelerated voltage of 5 kV and magnification of 20 k.

### 3.7. Analytical Methods

Free amino nitrogen (FAN) concentration in the hydrolysates and fermentation samples were determined by the ninhydrin colorimetric method [54]. After the end of fermentations, BC was removed from the culture broth and rinsed with water. Subsequently, BC was treated with 2 M NaOH to remove bacterial cells and then washed repeatedly until a neutral pH was obtained. BC was air dried at 35 °C until constant weight and finally weighed. The concentration of carbon sources were determined by High Performance Liquid Chromatography (Prominence, Shimadzu, Kyoto, Japan) equipped with an Aminex HPX-87H (BioRad, Hercules, CA, USA) column, coupled to a differential refractometer (RID-10A, Shimadzu, Kyoto, Japan). The mobile phase was a 10 mM H_2_SO_4_ aqueous solution with 0.6 mL/min flow rate. All analyses were performed in triplicate.

## 4. Conclusions

The present study showed that the bacterial strain *K. sucrofermentans* DSM 15973 can produce high BC concentrations when it is cultivated in by-product streams from oilseed-based biodiesel industries and waste streams from confectionery industries as the sole source of nutrients. The renewable resources employed provided all nutrients required for bacterial growth and BC production. This approach could lead to improved cost-competitiveness of industrial BC production. The properties of the BC obtained from the crude renewable resources compared well with literature-cited publications and the properties of BC produced with commercial nutrient supplements. Future research should focus on the identification of specific applications for the BCs produced from the crude renewable resources employed in this study.
